# Pan-enteric Video Capsule Endoscopy in Hereditary Haemorrhagic Telangiectasia: Gastrointestinal Manifestations and Feasibility

**DOI:** 10.1055/a-2925-5291

**Published:** 2026-08-11

**Authors:** Pernille Darre Haahr, Anton Bilenko, Jens Kjeldsen, Simon Tholander, Mikael Kjær Poulsen, Annette Dam Fialla, Anette Drøhse Kjeldsen

**Affiliations:** 1 Department of Oto-rhino-laryngology 11286 Odense University HospitalOdenseDenmark; 2 Department of Medical Gastrointestinal Diseases 11286 Odense University HospitalOdenseDenmark; 3 VASCERN HHT Reference Centre 11286 Odense University HospitalOdenseRegion SyddanmarkDenmark; 4 Department of Cardiology 11286 Odense University HospitalOdenseDenmark

**Keywords:** hereditary haemorrhagic telangiectasia, telangiectasia, video capsule endoscopy, pan-enteric

## Abstract

**Background and aims** Gastrointestinal telangiectasias are a recognised manifestation of hereditary haemorrhagic telangiectasia (HHT), but data on their nature and distribution throughout the entire gastrointestinal tract are limited. We aimed to evaluate gastrointestinal disease manifestations using pan-enteric video capsule endoscopy (VCE) and to assess its applicability in a non-selected group of patients with HHT.

**Methods** In this cross-sectional study, patients aged ≥50 years with confirmed HHT residing in the Region of Southern Denmark were invited to undergo pan-enteric VCE. Telangiectatic lesions were recorded according to anatomic location and lesion burden. Feasibility outcomes included patient acceptance, completion rate, mucosal visibility and transit times. Associations between active gastrointestinal bleeding and haemoglobin concentration were analysed using multivariable linear regression.

**Results** Of 52 eligible patients, 28 underwent VCE. One examination was lost due to technical failure, leaving 27 recordings available for analysis. Telangiectatic lesions were identified in 96.3% of evaluable examinations and were predominantly located in the stomach (74%) and the proximal small bowel (81.5%). Active bleeding was observed in 14.8% of patients, only in the upper gastrointestinal tract, and was independently associated with lower haemoglobin concentrations (β −2.84 g/dL, 95% CI −4.69 to −1.00; *p* = 0.004). Seven examinations (25.9%) were incomplete, and colonic mucosal visibility was frequently suboptimal. No capsule retention or procedure-related complications occurred.

**Conclusion** Pan-enteric VCE demonstrated widespread gastrointestinal involvement in HHT, predominantly in the proximal gastrointestinal tract. Although active bleeding was infrequent, it was associated with lower haemoglobin levels. Practical limitations suggest that the role of pan-enteric VCE in routine evaluation of HHT requires further clarification.

## Highlights

- Gastrointestinal telangiectasias were identified in 96% of patients with HHT undergoing pan-enteric video capsule endoscopy.
- Lesions were predominantly located in the stomach and proximal small bowel, whereas colonic involvement was less reliably visualised.
- Active gastrointestinal bleeding was detected in a minority of patients but independently associated with lower haemoglobin concentrations.
- Pan-enteric VCE enabled comprehensive assessment of gastrointestinal involvement, although limitations in patient acceptance, completion rate and colonic visibility affect its clinical applicability.

## Background

Hereditary haemorrhagic telangiectasia (HHT) is a rare systemic vascular disorder characterised by abnormal blood vessel formation. The disease is caused by loss-of-function germline mutations, often accompanied by a second-hit somatic mutation, in genes involved in the transforming growth factor-beta (TGF-β) signalling pathway. 1 2 3 4 5 Dysregulation of this pathway results in impaired angiogenesis and vasculogenesis, leading to the development of fragile vascular malformations, known as telangiectasia or arteriovenous malformations. HHT is inherited in an autosomal dominant manner and has an estimated prevalence of 1–2 per 10,000 individuals. 6 7

Gastrointestinal involvement is a recognised manifestation of HHT and represents an important cause of chronic blood loss, particularly in patients over the age of 50 years. 8 9 Gastrointestinal bleeding may occur in combination with epistaxis or as an isolated manifestation and may lead to iron deficiency and iron deficiency anaemia. Telangiectatic lesions have been reported throughout the gastrointestinal tract; however, their distribution and characterisation remain incompletely characterised. Previous studies suggest a predominance in the upper gastrointestinal tract and small bowel, with reported prevalences ranging from 29% to 64% in the stomach and 81–91% in the small bowel. 10 11 12 13

In contrast, data regarding the colonic involvement are limited and inconsistent. Some studies report large bowel involvement in approximately 30% of patients, whereas others suggest substantially lower frequencies, 13 14 Interpretation is complicated by the difficulty in distinguishing HHT-related telangiectasias from sporadic angiodysplasias, as well as by incomplete visualisation during conventional endoscopy. Consequently, the true pan-enteric distribution and burden of telangiectatic lesions in HHT remain uncertain.

Current guidelines recommend oesophagogastroduodenoscopy (OGD) as the initial diagnostic modality in patients with suspected gastrointestinal bleeding, followed by small bowel video capsule endoscopy (VCE) and/or colonoscopy when upper endoscopy does not adequately explain clinical findings. 15 However, these investigations are typically performed sequentially and assess the gastrointestinal tract in segments. Pan-enteric capsule systems, originally developed for inflammatory bowel disease, enable non-invasive visualisation of the entire gastrointestinal tract during a single examination. The utility, diagnostic yield and feasibility of such systems in patients with HHT have not been systematically evaluated.

The primary aim of this study was to characterise the prevalence, anatomic distribution and burden of gastrointestinal telangiectatic lesions throughout the gastrointestinal tract using pan-enteric VCE. A secondary aim was to assess the feasibility and clinical applicability of pan-enteric capsule endoscopy as a diagnostic tool in patients with HHT.

## Method

This cross-sectional study included patients with HHT who were eligible for pan-enteric VCE. Eligibility criteria were age ≥50 years, residence in the Region of Southern Denmark, confirmed diagnosis of HHT according to the Curaçao criteria and/or genetic testing, absence of known inflammatory or obstructive bowel disease and clinical suitability to undergo bowel preparation and capsule examination.

Eligible patients were identified through the Danish HHT Database, which includes both symptomatic patients and asymptomatic relatives diagnosed through clinical assessment and genetic screening. The database is maintained by the Danish HHT Centre of Excellence, which has served as the national referral centre for HHT since the early 2000s.

All eligible patients were invited to participate and were contacted by personal mail followed by telephone contact. Written informed consent was obtained from all participants prior to any study procedures. Patients who consented underwent clinical evaluation and pan-enteric VCE.

The study was approved by the Regional Danish Ethics Committee (ID S-20210037).

### Video Capsule Endoscopy

Pan-enteric VCE was performed using the Medtronic PillCam™ Crohn’s capsule system (Medtronic, Dublin, Ireland). The capsule is equipped with dual cameras providing a 172-degree field of view and adaptive frame-rate technology (4–35 frames per second), enabling dynamic image acquisition according to the capsule movement throughout the gastrointestinal transit.

Patients were instructed to discontinue oral iron supplementation, high-fibre or grain-rich diets and medications known to affect gastrointestinal motility during the week preceding the examination. Bowel preparation consisted of a clear-liquid diet on the day preceding the examination, followed by administration of Plenvu® and 2 mg prucalopride (Resolor) 45–60 min before capsule ingestion. After overnight fasting, the capsule was swallowed with water in the morning and followed by per oral ingestion boosters. The preparation regimen was selected in accordance with the standard protocol used for pan-enteric capsule examinations at our institution.

All VCE recordings were reviewed by a single senior gastroenterologist, blinded to clinical and biochemical data. The single-reader approach was used to standardize lesion identification and counting across segments and to reduce interobserver variability. The reviewer has completed formal basic and advanced capsule endoscopy training and has >12 years of tertiary-centre capsule diagnostics experience, including second-opinion reviews for referring hospitals. Capsule completeness, regional transit times and bowel cleanliness were recorded. Bowel cleanliness was assessed subjectively by the reviewing gastroenterologist and categorised as poor (no diagnostic yield), OK, good or excellent.

Telangiectasia is defined as a permanent dilatation of small blood vessels within the mucosa. 16 In HHT, telangiectasias represent focal small arteriovenous malformations (micro-AVMs) characterised by direct arteriovenous connections without an intervening capillary bed.

On capsule endoscopy, telangiectatic lesions were defined as well-demarcated, bright red, flat or slightly elevated mucosal vascular lesions, consistent with previously described vascular ectasias or angioectasias. 17

Telangiectatic lesions were identified and categorised according to anatomic location (oesophagus, stomach, proximal third of small intestine, middle third of the small intestine, distal third of the small intestine, right colon, transverse colon and left colon). Burden of lesions within each segment was categorised according to number (0, 1–5, 6–10 or 11+ lesions). The presence and anatomic localisation of active bleeding were documented.

### Clinical Evaluation

All participants underwent a structured clinical assessment, including a medical interview addressing comorbidities and current and previous medication use. Blood samples were obtained and analysed for haemoglobin, ferritin and other relevant biochemical parameters. Epistaxis severity was assessed using the validated Epistaxis Severity Score (ESS), which evaluates the frequency, duration and intensity of nosebleeds. 18 Iron deficiency was defined as ferritin <30 μg/L. Iron deficiency anaemia was defined according to World Health Organization criteria as haemoglobin <12 g/dL in women and <13 g/dL in men in the presence of iron deficiency. 19

### Statistics

This was a cross-sectional study with a primarily descriptive design. Continuous variables were summarised as mean ± standard deviation (SD) for normally distributed data and median with interquartile range (IQR) for non-normally distributed data. Categorical variables were presented as absolute numbers and percentages.

The primary outcome was the prevalence of gastrointestinal telangiectasias, calculated as proportions with corresponding 95% confidence intervals (CIs). Lesion distribution according to anatomic location and lesion burden (0, 1–5, 6–10, ≥11 lesions) was summarised descriptively. The association between gastrointestinal bleeding detected on VCE and haemoglobin concentrations was analysed using linear regression, adjusting for epistaxis severity, age and sex.

Secondary outcomes assessing feasibility of pan-enteric VCE, including capsule completion rate, bowel cleanliness and regional transit times and evaluation times were reported descriptively.

All tests were two-sided, and a *p* -value < 0.05 was considered statistically significant. Statistical analyses were performed using Stata/BE, version 19 (StataCorp, College Station, TX, USA).

## Results

### Study Population

A total of 52 patients met the eligibility criteria, of whom 28 consented to participate and were included between October 2025 and January 2026 at Odense University Hospital. All included patients had a confirmed diagnosis of HHT based on the fulfilment of the Curaçao criteria and/or genetic confirmation in the context of a supportive family history.

Among the 24 patients who declined participation, 18 cited the bowel preparation as the primary reason, while five declined due to lack of time and one initially consented but discontinued due to vomiting during bowel preparation. Baseline characteristics of participants and non-participants are shown in **Supplementary Table 1** . No significant differences were observed between groups with respect to age, sex, genotype distribution, ESS, haemoglobin concentration or prevalence of anaemia.

The final cohort was comprised of 16 women (57.1%) and 12 men (42.9%), with a mean age of 62 ± 10.0 years (range 50–82). Of the patients who declined participation, 13 (54.2%) were women and 11 (45.8%) were men and the mean age was 66.9 ± 10.7 (range 50–84). Baseline clinical and laboratory characteristics of the VCE cohort are presented in Table 1 .

**Table 1:** Baseline characteristics of the study cohort and the VCE subgroup. Data are presented as mean (standard deviation) unless otherwise stated.

	VCE *n* = 28
Age, years	62.0 (10.0)
Female sex, *n* (%)	16 (57.1)
Body mass index, kg/m ^2^	26.1 (4.8)
ESS score, median (IQR)	2.8 (1.4–4.5)
Iron deficiency, *n* (%)	6 (22.2)
Iron deficiency anaemia, *n* (%)	10 (37.0)
***Medication at time of study***	
*Bevacizumab, n (%)*	3 (10.7)
*Intravenous iron supplement, n (%)*	3 (10.7)
*Oral iron supplement, n (%)*	14 (50.0)
***Laboratory findings***	
Haemoglobin, g/dL	13.2 (2.1)
Ferritin, μ/L, median (IQR)	51 (31–68)
Transferrin (g/L)	33.8 (4.6)
Reticulocytes ×10 ^9^ /L	81.4 (29.5)
CRP, mg/L, median (IQR)	1.3 (0.6–2.3)

At baseline, six patients (22.2%) had iron deficiency and ten (37.0%) met the criteria for iron deficiency anaemia.

#### *Gastrointestinal Disease Manifestation*

Of the 28 patients who underwent VCE, one examination was lost due to technical failure. The remaining 27 recordings were completed and available for analysis.

**Fig. 1:**
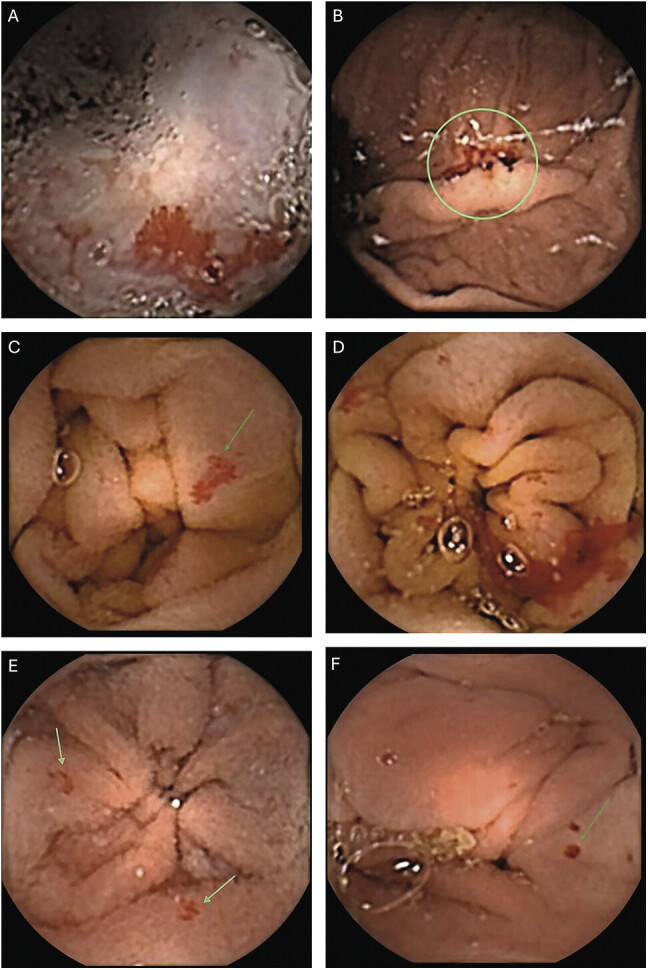
Representative findings on pan-enteric capsule endoscopy. ( **A** ) Oesophageal telangiectasia. ( **B** ) Gastric telangiectasia. ( **C** ) Small bowel telangiectasia. ( **D** ) Multiple small bowel telangiectasias with active bleeding. ( **E** ) Multiple small bowel telangiectasias. ( **F** ) Colonic telangiectasia.

Telangiectatic lesions were identified in 26 of 27 patients (96.3%, 95% CI 76–99%). Lesions were predominantly located in the proximal gastrointestinal tract, with a prevalence of 74% (95% CI 55.3–86.8) in the stomach and 81.5% (95% CI 63.3–91.8) in the proximal small bowel ( Table 2 ). Representative examples of telangiectatic lesions are shown in Fig. 1 .

**Table 2:** Distributions of telangiectatic lesions and bleeding by gastrointestinal segment.

Segment	Distribution of telangiectatic lesions				Bleeding ( *n* , % of examinations)
	No lesions	1–5	6–10	11+	
Oesophagus	25	2	0	0	0
Stomach	7	19	1	0	2 (7.4%)
Proximal small bowel	5	14	2	6	2 (7.4%)
Mid small bowel	12	11	2	2	0
Distal small bowel	10	12	2	3	0
Right colon	16	11	0	0	0
Transverse colon	22	5	0	0	0
Left colon	20	7	0	0	0

Values represent number of examinations. Percentages refer to the proportion of examinations with visible bleeding within each segment.

Most patients had a limited lesion burden (1–5 lesions) in each segment. However, in patients with more extensive disease (≥6 lesions), lesions were primarily located in the small bowel. Specifically, ≥6 lesions were observed in eight patients in the proximal small bowel, four in the mid small bowel and five in the distal small bowel.

**Fig. 2:**
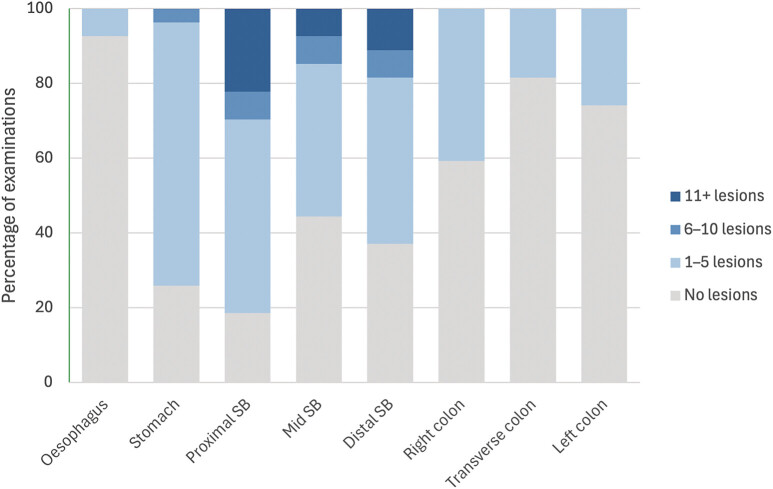
Distribution and burden of gastrointestinal telangiectatic lesions by anatomic segment. Stacked bars represent the proportion of examinations within each gastrointestinal segment according to lesion burden category (0, 1–5, 6–10 and 11+ lesions).

Of the 26 patients with telangiectatic lesions, 24 (92%) had lesions in more than one segment of the gastrointestinal tract. The anatomic distribution and burden of telangiectatic lesions across gastrointestinal segments are shown in Fig. 2 . Lesions were most frequently observed in the stomach and proximal small bowel, whereas colonic involvement was less common and generally associated with a low lesion burden.

Active bleeding was identified in four examinations (14.8%), exclusively in the stomach and the proximal small bowel. The patients with gastric bleeding had 1–5 telangiectatic lesions, whereas those with small bowel bleeding exhibited a higher lesion burden (≥11 lesions) in the proximal small bowel.

In multivariate linear regression analysis, gastrointestinal bleeding detected on VCE was independently associated with lower haemoglobin levels (β −2.84 g/dL, 95% CI −4.69 to −1.00; *p* = 0.004), after adjustment for ESS, sex and age. Male sex was, as expected, independently associated with higher haemoglobin concentrations (β 2.18 g/dL, 95% CI 0.91–3.44; *p* = 0.002), whereas ESS score and age were not significantly associated with haemoglobin concentration in this model.

No association was observed between haemoglobin concentration and the overall burden of gastrointestinal telangiectasias detected on capsule endoscopy (Spearman’s rho = −0.03, *p* = 0.846). Similarly, telangiectasia burden in the stomach and proximal small bowel was not associated with haemoglobin concentration (β = −0.18, 95% CI −0.79 to 0.43, *p* = 0.549).

#### *Applicability of Pan-enteric VCE*

The acceptance rate among eligible patients was 54%, with bowel preparation being the primary reason for refusal.

Seven out of 27 studies (25.9%) failed to reach completion as the video capsule reached the end of its operational battery life. Recording ceased in the right colon ( *n* = 1), left colon ( *n* = 2) and rectum ( *n* = 4).

**Fig. 3:**
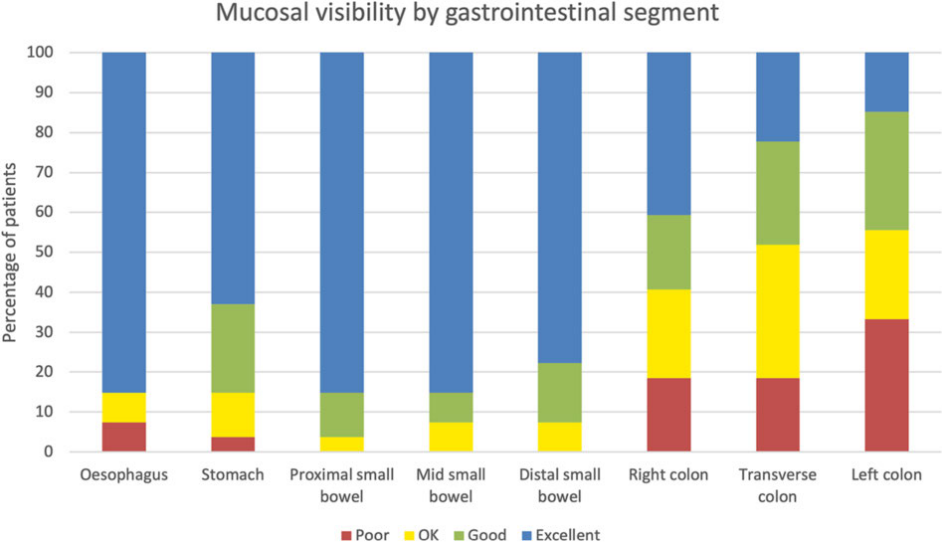
Mucosal visibility by gastrointestinal segment. Bars represent the percentage of patients in each visibility category (poor, OK, good, excellent).

Mucosal visibility was predominantly rated as good or excellent in the oesophagus, stomach and small bowel. In contrast, visibility in the colonic segments was more heterogeneous. Visibility was considered insufficient for diagnostic interpretation in 18.5% of examinations in the right and transverse colon and in 33.3% of examinations in the left colon ( Fig. 3 ).

The median gastric transit time was 20 min (IQR 13–34 min), and the median small bowel transit time was 82 min (IQR 44–121 min). The colonic transit time was a median of 3.8 h (IQR 2.8–9.7 h).

No cases of capsule retention or procedure-related complications were observed.

The mean reading time for video interpretation was 43.8 ± 18.2 min.

## Discussion

This is, to our knowledge, the first study to evaluate gastrointestinal telangiectatic lesions throughout the entire gastrointestinal tract using pan-enteric VCE in patients with HHT. Telangiectasias were identified in nearly all patients examined (96.3%) and were predominantly located in the proximal gastrointestinal tract, with prevalences of 74% in the stomach and 81% in the proximal small bowel. These findings are consistent with previous reports demonstrating that gastrointestinal telangiectasias in HHT are primarily located in the upper gastrointestinal tract, with reported frequencies ranging from 29% to 92%. 10 11 12 13

Furthermore, most patients had telangiectatic lesions in several gastrointestinal segments, supporting the widespread and systemic nature of the disease. This distribution likely reflects the diffuse vascular dysregulation characteristic of HHT.

Active gastrointestinal bleeding was observed in a minority of patients (14.8%) and confined to the stomach and proximal small bowel. Gastric bleeding did not appear to correlate with lesion burden, whereas small bowel bleeding was primarily associated with a higher telangiectatic lesion burden within the intestinal mucosa. Interestingly, haemoglobin concentration was not associated with either the overall burden of gastrointestinal telangiectasias or the burden of lesions in the stomach and proximal small bowel. These findings suggest that lesion burden alone may not be the principal determinant of anaemia in HHT.

Bleeding detected on VCE was associated with lower haemoglobin concentrations after adjustment for epistaxis severity, age and sex. However, only four patients had active bleeding at the time of VCE, and this finding should therefore be interpreted cautiously. Notably, a greater proportion of patients fulfilled the criteria for iron deficiency anaemia than had evidence of active gastrointestinal bleeding at the time of examination. This discrepancy may reflect the intermittent nature of gastrointestinal bleeding in HHT. The absence of visible bleeding during capsule examination therefore does not exclude clinically relevant blood loss over time. Taken together, these findings suggest that the clinical impact of gastrointestinal involvement in HHT may depend more on intermittent bleeding activity than on the extent of visible telangiectatic disease.

The relatively low ESS scores, indicates predominantly absent or mild epistaxis in this cohort, together with the observed association between VCE-detected bleeding and lower haemoglobin concentrations, suggest that gastrointestinal bleeding may contribute to anaemia in some patients with HHT. However, this observation should be interpreted cautiously given the small number of bleeding events.

Pan-enteric VCE was performed in 51.9% of eligible patients, with bowel preparation constituting the primary barrier to participation. Among completed examinations, the detection rate of telangiectatic lesions was high (96.3%), reflecting the frequent gastrointestinal involvement in HHT, particularly in patients aged >50 years. However, feasibility considerations should be considered when interpreting these findings. A quarter of examinations were incomplete (25.9%), and mucosal visibility in the colon was frequently suboptimal, limiting diagnostic confidence in this location. Reported completion rates in previous capsule studies have ranged from 69% to 100%, placing the present results within the lower spectrum of published experiences. 20

Various bowel preparation regimens have been described in prior studies, most commonly polyethylene glycol-based protocols supplemented with booster doses following capsule ingestion, similar to the present study protocol. Reported rates of adequate bowel cleansing vary widely (29–90%), and the colonic visibility observed in the present study appears comparable to the lower-to-mid range of these reports. 20 The preparation regimen used in the present study reflected routine clinical practice at our institution and may have contributed to the variability in colonic visibility observed.

These findings suggest that while pan-enteric VCE is capable of identifying gastrointestinal telangiectasias with a high detection rate, particularly in the upper gastrointestinal tract and small bowel, assessment of colonic involvement remains uncertain. The relatively high proportion of incomplete examinations and suboptimal colonic visibility may have reduced the detection of colonic lesions. Consequently, the low prevalence of colonic telangiectasias observed in the present study should be interpreted with caution, and negative colonic findings cannot be considered evidence of absence of disease.

Given that the majority of telangiectatic lesions, and all observed active bleeding, were located in the stomach and proximal small bowel, our findings suggest that conventional OGD may be sufficient for the initial evaluation of some patients with HHT. However, further studies directly comparing diagnostic modalities are needed before conclusions regarding the optimal diagnostic strategy can be drawn.

Although pan-enteric VCE demonstrated certain practical limitations, the present findings highlight that gastrointestinal bleeding in HHT may be clinically silent yet physiologically significant. Whereas epistaxis is typically apparent to the patient, gastrointestinal bleeding may go unrecognised while still contributing to chronic iron loss and anaemia. This underscores the importance of considering gastrointestinal evaluation in patients with unexplained anaemia despite mild epistaxis. However, future studies are warranted to clarify the optimal diagnostic strategy for gastrointestinal evaluation in this patient population.

Several limitations should be acknowledged. First, the cross-sectional study design precludes longitudinal assessment of disease progression and temporal relationships. This is particularly relevant in HHT, as both gastrointestinal bleeding and epistaxis may fluctuate over time, and iron parameters and haemoglobin concentrations are influenced by these dynamic bleeding patterns. Secondly, the study population was relatively small and restricted to patients aged ≥50 years, which may limit generalisability. Although all eligible patients were invited, participation was voluntary and only approximately half of eligible patients underwent VCE. Bowel preparation was the predominant reason for non-participation, which may have introduced selection bias by favouring individuals more willing to undergo extensive diagnostic procedures. However, baseline characteristics of participants and non-participants were broadly comparable with respect to age, sex, genotype distribution, epistaxis severity, haemoglobin concentration and anaemia prevalence ( **Supplementary Table 1** ), suggesting that substantial selection bias is unlikely. Third, VCE recordings were interpreted by a single experienced reader, and interobserver variability was not assessed. Although the single-reader approach ensured consistent lesion classification throughout the study, it precludes evaluation of reproducibility and may limit the generalisability of the findings, particularly for subtle vascular lesions and assessment of bowel cleanliness, which may be subject to observer variation. Lastly, 25.9% of examinations were incomplete and colonic visibility was frequently suboptimal, potentially reducing the detection of colonic lesions. Finally, only four patients had active gastrointestinal bleeding detected on VCE. Although a statistically significant association with lower haemoglobin concentration was observed, the small number of bleeding events limits the precision and robustness of the regression estimates. These findings should therefore be interpreted with caution and considered exploratory.

Despite the limitations, several strengths of this study should be emphasised. The cohort was derived from the national HHT reference centre with systematic registration in the Danish HHT database, ensuring well-characterised diagnoses and comprehensive clinical information. Although modest in size, the study population represents a broad clinical spectrum of HHT, including both symptomatic and asymptomatic individuals, thereby enhancing the clinical relevance of the findings. Importantly, this study provides, to our knowledge, the first systematic evaluation of the entire gastrointestinal tract using pan-enteric VCE in patients with HHT. The standardised examination protocol and blinded assessment by an experienced capsule endoscopist strengthen internal validity. Furthermore, the integration of endoscopic findings with biochemical parameters allows meaningful correlation between structural lesions and clinically relevant bleeding patterns. Finally, the study offers valuable real-world data on patient acceptance, completion rates and diagnostic limitations of pan-enteric VCE, which are essential for guiding further clinical implementation.

## Conclusion

In this cohort of patients with HHT, gastrointestinal telangiectasias were frequently identified and were predominantly located in the proximal gastrointestinal tract. Active bleeding was observed in a minority of patients but was associated with lower haemoglobin concentrations, suggesting potential clinical relevance. While pan-enteric VCE may offer comprehensive visualisation of gastrointestinal involvement, its practical limitations indicate that further studies are needed to clarify its role in the diagnostic evaluation of HHT.

## Supplementary Material

Ergänzendes Material Supplementary Material
