# Supplementary material for: Pan-enteric Video Capsule Endoscopy in Hereditary Haemorrhagic Telangiectasia: Gastrointestinal Manifestations and Feasibility
Source: Endosc Int Open. 2026 Aug 11;14:a29255291. doi: 10.1055/a-2925-5291 (PMC13463366; doi:10.1055/a-2925-5291)
Supplement: Supplementary file 1 [file 10-1055-a-2925-5291_29260107.pdf]

Supplementary Material

Pernille Darre Haahr, Anton Bilenko, Jens Kjeldsen, Simon Tholander, Mikael Kjær Poulsen, Annette Dam Fialla, Anette Drøhse Kjeldsen

Pan-enteric video capsule endoscopy in hereditary haemorrhagic telangiectasia: gastrointestinal manifestations and feasibility

Supplementary Table 1. Baseline characteristics of participants and non-participants eligible for pan-enteric video capsule endoscopy.

Data are presented as mean ± standard deviation (SD) or number (%), unless otherwise stated. P-values were calculated using Student's t-test for continuous variables and Pearson's chi-square test or Fisher's exact test for categorical variables, as appropriate.

Characteristic	Participants (n=28)	Non-participants (n=24)	p-value
Age (years) mean ± SD	61.7 ± 9.98	66.9 ± 10.6	0.077
Female sex, n (%)	15 (55.6)	13 (54.2)	0.921
Genotype, n (%)			0.385
HHT1	15	16	
HHT2	11	5	
JP-HHT	0	1	
No genotype HHT	1	2	
ESS, mean ± SD	3.03 ± 1.89	3.15 ± 1.87	0.830
Haemoglobin (g/dL), mean ± SD	13.2 ± 2.05	13.6 ± 1.94	0.587
Anaemia, n (%)	10 (37.0)	5 (20.8)	0.205

Abbreviation: ESS, Epistaxis Severity Score; HHT, hereditary haemorrhagic telangiectasia; JP-HHT, juvenile polyposis-hereditary haemorrhagic telangiectasia syndrome.
